# Participation and performance characteristics in half-marathon run: a brief narrative review

**DOI:** 10.1007/s10974-022-09633-1

**Published:** 2022-11-03

**Authors:** Pantelis Theodoros Nikolaidis, Beat Knechtle

**Affiliations:** 1 School of Health and Caring Sciences, University of West Attica, Ag. Spyridonos, 122 43 Egaleo, Athens Greece; 2 Institute of Primary Care, University of Zurich, Zurich, Switzerland

**Keywords:** Aerobic capacity, Anaerobic threshold, Endurance, Exercise, Nutrition, Participation, Running economy

## Abstract

Half-marathon (HM) is a running sport of increasing popularity in both sexes and in all age groups worldwide during the last years. Many studies have examined several aspects of HM, such as performance and participation trends, sex and age differences, physiological correlates, and training; however, no comprehensive review has ever been contacted to summarize the recently accumulated knowledge. Therefore, the aim of the present study was to review all previous research in this sport, focusing on participation and performance aspects. It was shown that HM runners had similar anthropometric and physiological characteristics as full-marathon runners which should be attributed to the affinity of these two races in terms of metabolic demands. Performance in HM was related with superior scores in aerobic capacity (maximal oxygen uptake, anaerobic threshold and running economy) and training characteristics (sport experience, weekly distance, training speed, frequency of sessions and long single endurance run distance), and lower scores in adiposity-related scores (e.g. body mass, body mass index, body fat percentage and skinfold thickness). Considering the popularity of HM race and the lack of many original studies (compared to FM race), this is an exciting field for scientific research with a large potential for practical applications, since the majority of HM runners are amateur runners in need of sex-, age- and performance-tailored exercise prescription.

## Introduction

Half-marathon (HM) is a running event of increasing popularity in both sexes and in all age groups worldwide during the last years (Bonet et al. 2022). Although full-marathon (FM) is the most popular endurance running distance, most runners participate in HM (Cribari et al. 2013). Many studies have examined several aspects of HM, such as performance and participation trends, sex and age differences, physiological correlates, and training,however, no comprehensive review has ever been contacted to summarize the accumulated knowledge. Therefore, the aim of the present study is to review all previous research covering all aspects such as epidemiological trends, the role of age and sex, especially focusing on the physiological aspects.

### Participation

**Fig. 1:**
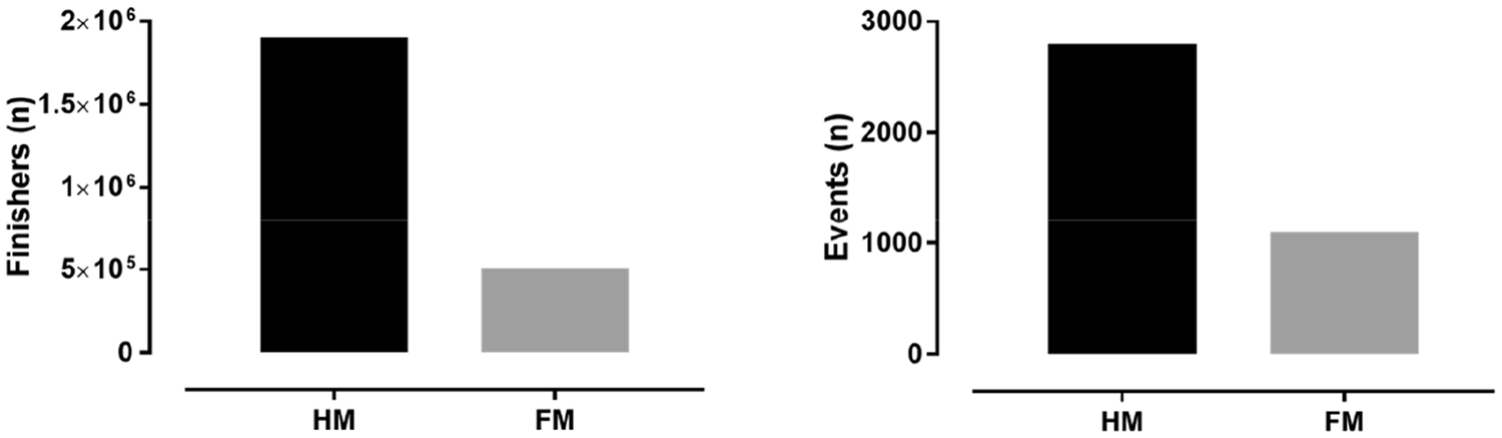
Finishers and events in USA in 2016. Source: http://www.runningusa.org (accessed on 16/9/2017)

Main aspects of participation included the numbers of participants in HM and whether these numbers would change across calendar years, sex differences in participation (typically examined using the men-to-women ratio) and age across years, whereas these aspects may vary by nationality. In 2016, more finishers and events were observed in HM than in FM (Fig. 1). Participation trends in HM have been examined with regards to FM in a single country, Switzerland, where those participating in HM are three times more than those competing in FM races (Anthony et al. 2014). It has been observed in 226,754 HM and 86,419 FMrunners competing in Switzerland between 2000 and 2010 that the number of HM increased from 2000 to 2010 for both men (+ 231%) and women (+ 299%), whereas the number of male and female FM runners increased until 2005 only and decreased thereafter (Anthony et al. 2014). A study on 508,108 runners (125,894 female and 328,430 male HM and 10,205 female and 43,489 male FM) competing between 1999 and 2014 in all flat HM and FM held in Switzerland showed that the number of women and men increased across years in both HM and FM, and there were 12.3 times more female HM than female FM and 7.5 times more male HM than male FM (Knechtle et al. 2016a). These different proportions of women and men competing in HM and FM races indicated that HM was a sport where relatively more women participated compared to FM. The abovementioned preliminary studies (Anthony et al. 2014; Knechtle et al. 2016a) highlighted a larger participation in HM than in FM races, and this trend was more striking in women than in men. An explanation of this discrepancy between sexes might be that women could be considered as more ‘novice’ runners than men, and consequently, should run more HM before entering FM races.

The participation and performance in HM may vary by nationality, and it would be interesting to focus on trends concerning East African runners who were considered as experts in long distance running events (Knechtle et al. 2016b).Actually, a study of ~ half million HM and FM runners originating from 126 countries and competing between 1999 and 2014 in all road-based HM and FM held in Switzerland reported that, in HM, 48 women (0.038%) and 63 men (0.019%) were from Ethiopia and 80 women (0.063%) and 134 men (0.040%) from Kenya, whereas in FM, three women (0.029%) and 15 men (0.034%) were from Ethiopia and two women (0.019%) and 33 men (0.075%) from Kenya (Knechtle et al. 2016b). These findings suggested that the largest percentage of participants in HM is of local origin.

In both women and men, the best performance in HM and FM held in Switzerland was achieved by East African runners with Ethiopian and Kenyan runners being the youngest in both sexes and formats of race (Knechtle et al. 2016b). These findings showed that East-African runners were the fastest in both HM and FM although they represented the smallest percentage of participants (Knechtle et al. 2016b). This observation was in agreement with an analysis of the world’s best HM runners during 1999–2015, where it was shown that most of them were Kenyans (30% in women and 57% in men) (Nikolaidis et al. 2017). According to the abovementioned effect of nationality, the characteristics of HM differed from country to country, e.g. the local people tended to participate more to races taking place in their country than foreigners. Furthermore, the participation and performance may change across years. Actually, a study examined the changes in participation, performance and age of East African runners competing in HM and FM held in Switzerland between 2000 and 2010 indicated that across time, the number of Kenyan and Ethiopian finishers remained stable while the number of Non-African finishers increased for both women and men in both HM and FM (Cribari et al. 2013). This difference in participation trends across years by nationality might be due to the increase of local ‘recreational’ runners, while the number of the most competitive runners coming from abroad would remain stable. To sum up, more runners compete in HM than in FM, and the fastest HM runners are East Africans.

### Age of peak performance

**Fig. 2:**
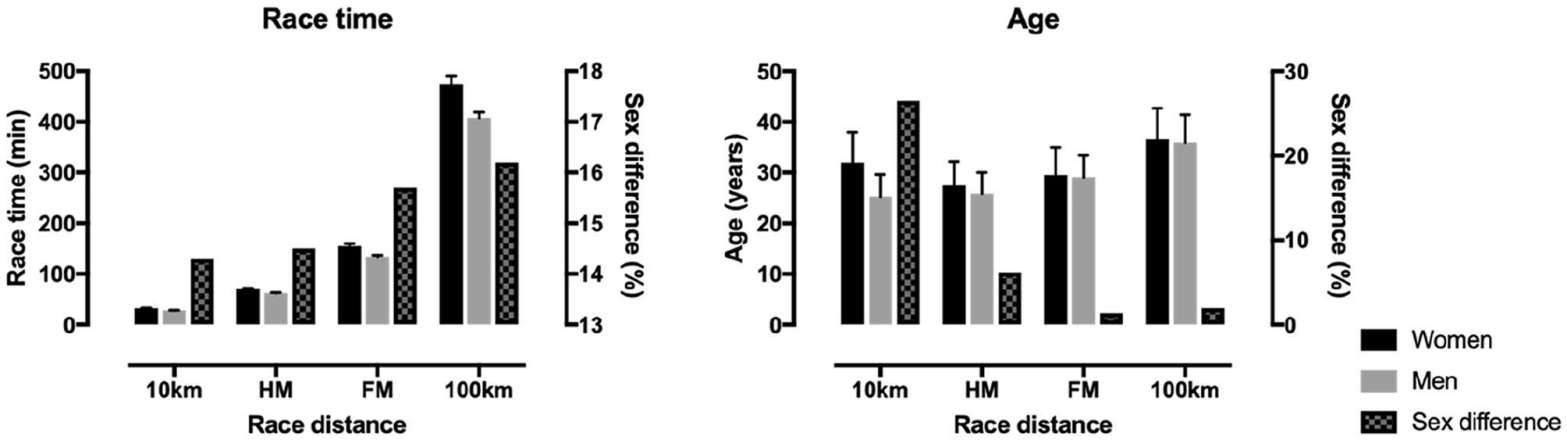
Race time and age of the best runners by race distance and sex. Based on Nikolaidis and colleagues Nikolaidis et al. 2017. Error bars represent standard deviations. HM = half-marathon, FM = full-marathon

Every sport has its own age of peak performance and thus, it would be important to estimate at which age HM runners achieve their peak performance in order to set long-term training goals. The largest part of the finishers in HM and FM held in Switzerland of both genders was assigned to age group 40–44 years in HM (19.5% of the total number of finishers) and FM (22.0% of finishers) (Anthony et al. 2014). For both HM and FM races, most of the female and male finishers were recorded in age group 40–44 years (Knechtle et al. 2016a; Knechtle and Nikolaidis 2018). In HM, women (41.4 years) were at the same age as men (41.3 years),in FM, women (42.2 years) were at the same age than men (42.1 years),however, women and men FM runners were older than their counterpart HM runners (Knechtle et al. 2016a). With regards to the age of peak performance, it may differ depending on the performance level, i.e. whether all or the top finishers were considered. For instance, in the world’s largest HM race—the GöteborgsVarvet—U40 was the fastest age group when all finishers were analyzed, whereas U35 were the fastest when the top 10 were considered (Knechtle and Nikolaidis 2018). Moreover, an analysis of the world’s best HM runners indicated an age of 26–27 years, which was younger than in FM and 100 km ultra-marathon races (Nikolaidis et al. 2017) (Fig. 2). This observation was confirmed by a study using non-linear regression on world records in HM, where the age peak performance was ~ 27 years (Nikolaidis et al. 2018).

The effect of age on HM performance differed from other endurance sports (Sterken 2005). It has been shown that age-related losses in endurance performance did not occur before the age of 50 years with mean FM and HM race times being identical for the age groups 20–49 years, whereas age-related performance decreases of the 50–69-year-old subjects were only in the range of 2.6–4.4% per decade (Leyk et al. 2007). These results suggested that the majority of older athletes were able to maintain a high degree of physical plasticity supporting the hypothesis that lifestyle factors had considerably stronger influences on functional capacity than the factor age (Leyk et al. 2007). No significant age-related decline in performance appeared before the age of 55 years, whereas only a moderate decline is seen thereafter (Leyk et al. 2010). Performance losses in middle age were mainly due to a sedentary lifestyle, rather than biological aging (Leyk et al. 2010). In summary, the average age of HM runners was 40–44 years, and the age of peak performance was younger than 35–40 years.

### Performance trends

Performance in HM might be examined using either race time or average speed. In HM held in Switzerland, women (10.29 ± 3.03 km/h) were faster than men (10.22 ± 3.06 km/h) as well as in FM, women (14.77 ± 4.13 km/h) were faster than men (14.48 ± 4.07 km/h) (Knechtle et al. 2016a). Slower HM race time by 13% (10% in FM) in women than in men was observed in a study of all and top 10 finishers aged 20–79 years (Leyk et al. 2007). Moreover, a sex difference of ~ 14% was noted in the world best HM runners (Nikolaidis et al. 2017). Sex differences in performance may also be attributed to the different adaptation to long-term exercise between women and men. A study on 16 males and 16 females preparing for a HM revealed a larger increase in the average daily metabolic rate in men than in women suggesting exercise stimulates more habitual physical activity and diet-induced thermogenesis in men than in women (Meijer et al. 1991). The variation of performance from a race to race seems to depend on competitive experience and attitude toward competing and was found 4.2% for the fastest quartile of men runners in HM with men, slower and younger runners presenting more variation (Hopkins and Hewson 2001). Physiological, anthropometric and training correlates of performance were examined in following sections,however, they might differ depending on performance level, considering that in the elite level other factors (e.g. shoe technology) would play an important role (Goss et al. 2022).

### Physiological correlates of performance

Physical fitness is classified as health- (consisting of body composition, aerobic capacity, muscular strength, muscular endurance and flexibility, i.e. components related directly to health) or sport-related (consisting of those components related to sport performance such as speed or reaction time). Based on the relatively long duration of a HM, it is reasonable to assume that performance in this sport relates to maximal oxygen uptake (VO_2_max), since it relies mostly on the aerobic energy transfer system. One of the oldest studies on HM (Williams and Nute 1983) already identified VO_2_max and anaerobic threshold as correlates of race time (r = −0.81 and r = −0.88, respectively). In addition, in male recreational runners, HM race time correlated with VO2max (r = −0.64), speed at VO2max (r = −0.84) and anaerobic threshold (r = −0.79) (Alvero-Cruz et al. 2019). These relationships were confirmed in HM runners with asthma, too,actually, the HM pace of asthmatic runners correlated largely with VO_2_max (r = 0.86) and almost perfectly with running speed at a blood lactate concentration of 2 mmol.L^−1^ (r = 0.97) (Freeman et al. 1990). In amateur runners (nine men, age 36 years), HM time was almost perfectly correlated with VO_2_max (r = 0.91) and speed corresponding to VO_2_max (r = 0.90) (Santos et al. 2012). Furthermore, a comparison among 400–800 m, 1500–3000 m and HM women runners showed that HM runners had the highest VO_2_max (Nurmekivi et al. 1998). In runners, the pace of HM was comparable to the maximal lactate steady state velocity (Legaz-Arrese et al. 2011).

Considering the physiological relevance of HM and FM races, previous research examined the relationship of performances in these two races (Salinero et al. 2017; Karp 2007; Coyle 2007). A research on 84 male amateur FM runners (aged 41.0 years, finish time 226.0 min) showed a very large correlation between FM and HM race time (r = 0.81) (Salinero et al. 2017). In 2004 U.S. Olympic Marathon Trials qualifiers (104 men, 151 women), FM performance correlated to HM performance (Karp 2007). Maintaining the world record pace for the HM in the FM would lead to run a FM in 1:58 h:min (Coyle 2007).

In addition to the abovementioned correlation studies, an approach to study determinants of performance in HM is to develop prediction equations of race time based on correlates (Pérez et al. 2012; Gómez-Molina et al. 2017), which include usually two steps, first, the development of an equation in a sample of runners, and second, the validation of this equation in another sample. For instance, a study on male runners considered training-related and anthropometric variables, and laboratory data from a graded exercise test (GXT) on a treadmill (VO_2_max, speed at the anaerobic threshold, peak speed) and biomechanical variables (contact and flight times, step length and step rate) (Gómez-Molina et al. 2017). This study found that HM race time could be predicted to 90.3% by variables related to training and anthropometry, 94.9% by physiological variables, 93.7% by biomechanical parameters and 96.2% by a general equation, and using these equations, the predicted time was significantly correlated with performance (r = 0.78, 0.92, 0.90 and 0.95, respectively) (Gómez-Molina et al. 2017). Moreover, HM race speed could be predicted as V21k (km/h) = (V2*1.085) + (−0.282*bLA2) −0.131, r2 = 0.97, ETE = 0.414 km/h, where V2 was the speed during a test in track at constant pace over 2400 m slightly higher than the competition expected pace and bLA2 blood lactate (Pérez et al. 2012). A research examined the ratio between running speed and heart rate (HR) as predictor for aerobic capacity (based on the assumption that lower HR at a given speed is expected for more fit individuals), and subsequently race time in 10 km, HM and FM (Altini et al. 2017). This study showed that the speed to HR ratio provides higher accuracy in aerobic capacity estimation compared to resting HR or no-physiological data, and large correlations between aerobic capacity and race time (r = 0.56–0.61) (Altini et al. 2017). In addition to the comparison with laboratory exercise testing, HM performance has been investigated with field tests such as Cooper test (Alvero-Cruz et al. 2019; Alvero-Cruz et al. 2020). For instance, Alvero-Cruz and colleagues (Alvero-Cruz et al. 2019; Alvero-Cruz et al. 2020) observed an almost perfect correlation between HM race time and Cooper test distance, and high predictive validity of this test.

Another methodological approach that may provide indirect information for the identification of correlates of performance is to examine the acute physiological responses to a HM by comparing values pre- and post-race (Dressendorfer 1991). For instance, runners were tested in a graded exercise test before and after a HM,time to exhaustion (6.0 vs 4.1 min), VO_2_max (60.0 vs 56.3 ml.kg^−1^.min^−1^), peak respiratory exchange ratio (RER; 1.18 vs 1.06), and peak La (9.7 vs 7.8 mmol.L^−1^) decreased after the HM (Dressendorfer 1991). Other studies focus on muscular acute responses to a HM (Boccia et al. 2017 Boccia et al. 2017).For instance, intermittent isometric rapid contractions of the knee extensor muscles were performed the day before and immediately after a HM, where it was observed that HM had a greater negative effect on repeated, rather than on single, attempts of maximal force production (Boccia et al. 2017). In another study of this research group that examined pre- and post-HM race maximum voluntary contractions of knee extensor muscles, it was found that post-race knee extensors showed a decreased strength (−13.9%) and a reduction in EMG amplitude (−13.10%) and in CV (−6.8%, p = 0.032) (Boccia et al. 2017). Moreover, compared to FM, a HM race induced smaller vertical jump height reduction and less self-reported muscle pain suggesting less muscular fatigue in HM than in FM race (Coso et al. 2017). The abovementioned studies (Boccia et al. 2017; Boccia et al. 2017; Coso et al. 2017) highlighted the importance of muscular fitness in addition to aerobic capacity for HM race. In line with the findings of the correlation studies presented in this section, a comparative study among different performance groups highlighted the role of physiological characteristics (Ogueta-Alday et al. 2018). Particularly, Oguea-Alday and colleagues (Ogueta-Alday et al. 2018) found that HM runners with race time 70 min had superior VO_2_max and running economy than those with race time 80 min, 90 min and 105 min. In summary, performance on HM race would depend mostly on VO_2_max and other indices of aerobic capacity.

### Anthropometric and training correlates of performance

Performance in HM is not related only to physiological characteristics, but also to anthropometry and training habits (Campbell 1985; Friedrich et al. 2014; Knechtle et al. 2012; Zillmann et al. 2013). For instance, a study in university HM runners observed that distance run per week and number of weeks training for the event, and body mass index (BMI) were predictors of HM race time (Campbell 1985). In female and male recreational HM runners, HM race time was related to body fat percentage (BF), running speed during training, and BMI was predictor of performance only in men (Friedrich et al. 2014), whereas elsewhere race time was more strongly associated with anthropometry in women than men (Knechtle et al. 2010). For male HM runners, BMI, BF and speed in running during training were related to race time (Zillmann et al. 2013). Furthermore, in female finishers of the ‘Half Marathon Basel’, race time was largely related to body mass, BMI, BF (r = 0.48–0.60), and could be predicted by the formula ‘166.7 + 1.7*midaxilla skinfold – 6.4*speed in training’(R^2^ = 0.71) (Knechtle et al. 2011). In HM, FM and UM master runners (> 35 years old), BF and training characteristics, not skeletal muscle mass, were associated with running times (Knechtle et al. 2012). A comparison between HM and FM men runners showed that HM runners were heavier, had longer legs, thicker upper arms, a thicker thigh, a higher sum of skinfold thicknesses, a higher body fat percentage and a higher skeletal muscle mass, fewer years of experience, completed fewer weekly training kilometers, and fewer weekly running hours (Zillmann et al. 2013). The relationship of HM with training characteristics might be explained by exercise-induced chronic cardiometabolic adaptations resulting in improvements in VO_2_max, a major predictor of performance (Meyler et al. 2021).

A comparative study between HM and FM races showed that a fast race time was associated with high weekly training volume (> 32 km) and a long training single distance (> 21 km) in HM, and high weekly training volume (> 65 km) in FM runners (Fokkema et al. 2020). Furthermore, a research among groups different performance groups confirmed the role of anthropometric and training characteristics on HM performance (Ogueta-Alday et al. 2018), where runners with race time 70 min had more sport experience and weekly running distance, and less body mass, BMI and skinfold thickness than those with race time 80 min, 90 min and 105 min. In summary, performance on HM race would depend mostly on VO_2_max and other indices of aerobic capacity. To sum up, HM performance depended on weekly running distance, number of training units, training running speed, BMI and BF (Campbell 1985; Friedrich et al. 2014; Knechtle et al. 2012; Zillmann et al. 2013). Therefore, a practical advice for recreational runners wishing to improve their race time might be to increase training (distance, running speed and frequency) and decrease BMI and BF.

## Pacing

Pacing in sport refers to the conscious or subconscious regulation of performance (e.g. speed during a race) in order to achieve a goal (Thiel et al. 2018; Micklewright 2019). Although pacing is related to performance in endurance sports (the less variable the pacing, the faster the race time), little information exist with regards to pacing strategies of HM runners (Hanley 2015). The relevant literature has been developed recently (Nikolaidis et al. 2019 Cuk et al. 2019; Cuk et al. 2020; Nikolaidis et al. 2019). A methodological approach to analyze pacing in HM has been to consider speed for intermediate segments, e.g. every 5 km (0–5, 5–10, 10–15 and 15–20 km) and end 1.1 km segments (Hanley 2015). An analysis of all finishers in Ljubljana 2017 HM showed a positive pacing with every segment being slower than its precedent one (Nikolaidis et al. 2019). In elite (finishers in the IAAF World Half Marathon Championships) HM runners, it was observed that the fastest finishers maintained split speed from 5 to 15 km, whereas slower runners decreased speed after 5 km (Hanley 2015). Compared to FM, an analysis of recreational HM runners observed a more even pacing in Ljubljana (Nikolaidis et al. 2019) and Vienna races (Cuk et al. 2020).A research of the pacing in the Great West Run HM showed that RPE scales with the proportion of exercise time that remains inferring that the brain uses a scalar timing mechanism (Faulkner et al. 2008). With regards to the variation of pacing by age, a research in Vienna City HM race revealed a positive pacing strategy, i.e. speed decreased across race, with an end spurt in all age groups and larger variation of speed in the younger age groups (Cuk et al. 2019). Additionally, a more even pacing was observed to relate with high training volume and long single training run (Fokkema et al. 2020). In summary, similarly to other endurance races, pacing was associated with performance in HM race with faster runners adopting a more even pacing than their slower counterparts. Thus, recreational runners would be recommended to maintain running speed during a HM race.

### Practical applications

With regards to the popularity of HM race, the findings of research on this field would concern an increasing number of HM runners. The fact that HM race has been a sport activity recently developed would imply that it could not be ‘covered’ by scientific and professional knowledge derived from long distance running – such as 5 km and 10 km – and FM race. This observation highlighted the need to develop further the research on this field. In this context, the present review attempted to address fundamental questions on the identity of HM runners (sex, age and nationality), performance trends and correlates, to develop practical information for professionals working with runners. For instance, the knowledge of the age of peak performance can aid setting long-term performance goals considering the age of runners. Several original research studies were identified that examined correlates of HM performance and enhanced our knowledge on this field. Based on these findings, practitioners working with HM runners should aim to optimize aerobic capacity (e.g., VO_2_max and anaerobic threshold), training indices (e.g., weekly running distance and running speed) and adiposity-related parameters. Although these parameters clearly were related to HM performance in recreational runners that might be considered as an heterogeneous group, a challenge for future studies would be to examine the variation of their importance depending on performance level.

## Conclusion

**Fig. 3:**
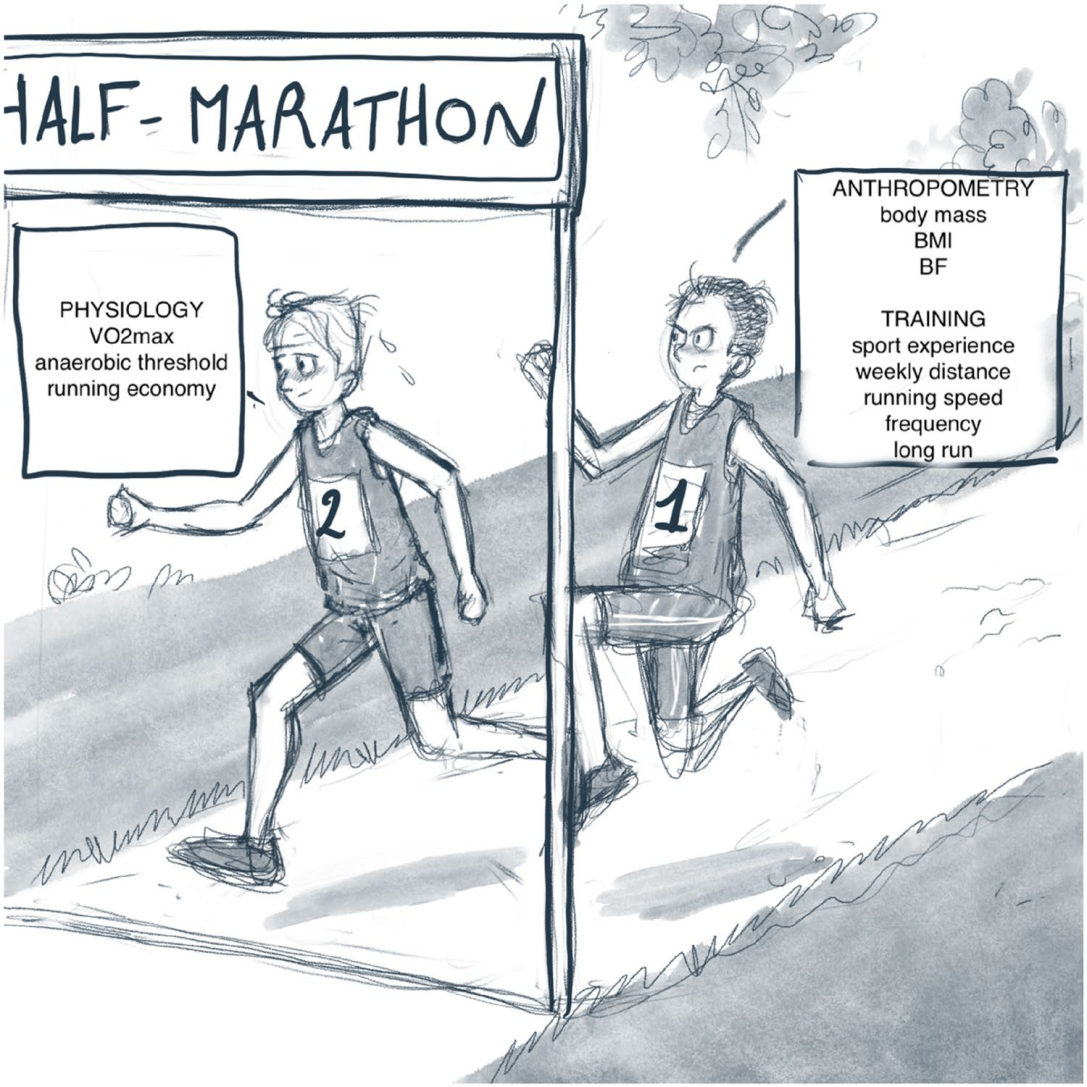
Physiological, anthropometric and training characteristics influencing half-marathon performance

In conclusion, performance in HM was related with superior scores in aerobic capacity (maximal oxygen uptake, anaerobic threshold and running economy) and training characteristics (sport experience, weekly distance, training speed, frequency of sessions and long single endurance run distance), and lower scores in adiposity-related scores (e.g. body mass, body mass index, body fat percentage and skinfold thickness) (Fig. 3). Considering the popularity of HM race and the lack of many original studies (compared to FM race), this is an exciting field for scientific research with a large potential for practical applications.

## Data Availability

All data are available by the corresponding author upon reasonable request.
